# Fabrication of Antimicrobial Multilayered Nanofibrous Scaffolds-Loaded Drug *via* Electrospinning for Biomedical Application

**DOI:** 10.3389/fbioe.2021.755777

**Published:** 2021-10-20

**Authors:** Qi Liu, Hengmin Jia, Wenchong Ouyang, Yan Mu, Zhengwei Wu

**Affiliations:** ^1^ School of Nuclear Science and Technology, University of Science and Technology of China, Hefei, China; ^2^ Department of Infection Control and Prevention, The First Affiliated Hospital of University of Science and Technology of China, Hefei, China; ^3^ CAS Key Laboratory of Geospace Environment, University of Science and Technology of China, Hefei, China

**Keywords:** multilayered nanofibers, drug delivery, poly(vinyl alcohol), antimicrobial, biodegradable

## Abstract

Nanofibers prepared by biobased materials are widely used in the field of biomedicine, owing to outstanding biocompatibility, biodegradable characters, and excellent mechanical behavior. Herein, we fabricated multilayered nanofibrous scaffolds in order to improve the performance of drug delivery. The composite layer-by-layer scaffolds were incorporated by hydrophobic poly(l-lactic acid) (PLA): polycaprolactone (PCL) and hydrophilic poly(vinyl alcohol) (PVA) nanofibers *via* multilayer electrospinning. Morphological and structural characteristics of the developed scaffolds measured by scanning electron microscopy (SEM), and transmission electron microscopy (TEM) confirmed smooth and uniform fibers ranging in nanometer scale. The differences in contact angles and Fourier transform infrared spectrum (FTIR) between single-layered PVA nanofibers and multilayered scaffolds verified the existence of PLA: PCL surface. *In vitro* biodegradable and drug release analysis depicted multilayered scaffolds had good biodegradability and potential for medical application. Due to the model drug incorporation, scaffolds exhibited good antibacterial activity against *Escherichia coli* and *Staphylococcus aureus* by the zone of inhibition test. These results revealed that the multilayered scaffolds were proved to be desirable antibacterial materials for biomedical application.

## Introduction

The struggle between humans and bacteria has lasted for thousands of years. Millions of patients have been afflicted with health and life-threatening bacterial diseases per year. Bacterial infections take a long time to cure, and both chronic and acute bacterial infections have always been a main clinical problem (Kim et al., 2015; Alves et al., 2016). Advanced materials with effective antimicrobial activity have great demand in the field of medical application (Hariu et al., 2017; Li et al., 2020). Variety of materials release loaded antibacterial agents sustainably, thus affording antimicrobial activity while maintaining a healthy concentration for tissue rehabilitation. So far, several kinds of antibacterial materials have been developed including hydrogel, electrospun nanofibers, and metal oxide (Zhao et al., 2017; Ebrahimzadeh et al., 2021; Ghiasi et al., 2021). Among these materials, electrospun nanofibers exhibit better porosity and mechanical properties to absorb tissue exudates and allow air to permeate, leading to wound recovery. Especially, electrospun nanofibers have excellent adaptability, such as *in situ* encapsulating drugs, tissue engineering, and cancer treatment (Wu et al., 2021; Yue et al., 2021).

The antibacterial materials need to mimic the natural extracellular matrix (ECM) to obtain good biocompatibility since it is in direct contact with tissue. The multilayered electrospun nanofibrous scaffold is a promising multifunctional structure, which is fabricated by a layer-by-layer pattern. Therefore, some related research studies about the characterization of such multilayered nanofibrous scaffolds that could promote cell adhesion, proliferation, and migration have already been reported. Researchers have proposed strategies to promote tissue regeneration such as three-layered stable scaffolds (Pattanashetti et al., 2020), 3D buckled scaffolds (Navaneethan et al., 2021), and graded biomimetic nanofibrous scaffolds (Hejazi et al., 2021). Multilayered scaffolds were also applied for the buccal drug delivery system (Nageeb El-Helaly et al., 2021) and controlled drug release (Charpashlo et al., 2021). Our study aimed to prepare a drug-loaded nanofibrous scaffold as a tool to enhance bioavailability and utilization with reduced potential hazards to the patient.

For biomedical applications, the choice of appropriate materials is critical. Polylactic acid (PLA) and polycaprolactone (PCL) is U.S. Food and Drug Administration (FDA)–approved materials for biomedical procedures. Pagno et al. assembled PBAT/PCL membranes to remove drug in environmental engineering (Pagno et al., 2020). Rafiei et al. fabricated 3D fibrous PCL scaffolds for protein delivery to mimic the characteristics of cartilage tissue (Rafiei et al., 2020). However, the hydrophobicity and mechanical behavior limit the application for drug loading and release. Polyvinyl alcohol (PVA) was selected as a synthetic polymer to improve the performance of PLA and PCL. PVA has attracted wide attention owing to non-toxic, hydrophilicity, and biocompatibility. A series of previous studies have been reported on tissue engineering, drug delivery, food industry, and environmental application (Kadokawa 2016; Kang et al., 2018; Kchaou et al., 2021). Excellent hydrophilicity enables PVA to encapsulate and release drug, which is revealed by our previous research (Liu et al., 2021). Accordingly, the purpose of this research meant to manufacture antibacterial materials with pretty stability and biodegradability by designing the multilayered nanofibrous structures of PLA, PCL, and PVA.

In this article, we prepared diverse multilayered nanofibrous scaffold-loaded moxifloxacin hydrochloride (MH) and silver sulfadiazine (SSD) for the drug loading system. The influence of the composition of various components on the scaffold was analyzed. Moreover, different physicochemical properties such as morphology of nanofibrous scaffolds, FTIR, porosity, water contact, biodegradability, drug release, and tensile test were was carried out. Finally, two model drugs were loaded with samples to analyze the drug-loading performance. Evaluation of antibacterial activity was studied by the agar disc diffusion method with Gram-positive and Gram-negative bacteria.

## Materials and Methods

### Materials

PVA (polymerization degree = 1700; alcoholysis degree = 88%) and PCL (M_w_ ∼ 80,000) were purchased from Qingdao Pansi Technology Co., Ltd. PLA (M_w_ ∼ 160,000) was purchased from NatureWorks LLC (United States). Organic solvents chloroform and dimethylformamide (DMF) were supplied by Sinopharm Chemical Reagent Co., Ltd. MH (the purity was 98%) and SSD (the purity was 98%) were purchased from Shanghai Macklin Biochemical Co., Ltd. The solvents of PVA are deionized water. All chemicals and solvents were used without further purification.

### Precursor Solutions Preparation

The blend solution of PLA (0.96 g) and PCL (0.24 g) was dissolved in a 10 ml solvent mixture of chloroform and DMF (8:2) under constant magnetic stirring at room temperature to obtain homogeneous PLA/PCL solution (12% w/v). The PVA solution was prepared by dissolving 12% w/v PVA in deionized water. To analyse the drug loading of the scaffold, the MH and SSD were added to the PVA solution with 50 mg per 10 ml. Each solution was prepared before electrospinning to ensure freshness.

### Multilayer Electrospinning

All the prepared precursor solutions were poured into a 10-ml plastic syringe. The electrospinning setup consists of a syringe pump (TYD01-01, Lead Fluid Technology Co., Ltd., China), high voltage power supply (Qingdao Pansi Technology Co., Ltd.), and a collector wrapped with aluminum foil. The procedure of multilayer electrospinning was as follows. MH and SSD were used as a model drug to investigate drug delivery and release performance of the multilayered scaffolds. The blend of PLA:PCL was electrospun as a first layer, the drug-loaded PVA was set as a second layer, and a third layer of PLA: PCL was again electrospun over PLA: PCL/PVA designated as PLA:PCL/PVA/PLA: PCL scaffolds. The consumption volume of the precursor solution of each nanofiber layer is controlled at about 2 ml. Finally, all the samples were dried in vacuum at 40°C for 24 h to remove residual solvents for further testing. Details of the electrospinning condition of all prepared samples were labeled, as shown in Table 1.

**TABLE 1:** Parameters of electrospinning condition of each sample.

Sample	Process parameters		
	Flow rate (μl/min)	Vol/kv	Distance (cm)
PVA	5	12	12
PLA:PCL	15	11.5	12
ML-S	15/5/15	11.5/12/1.5	12

### Characterization

Morphological characterization of nanofibrous scaffolds was performed by field emission scanning electron microscopy (SU8220, Hitachi, Japan) and transmission electron microscopy (JEM-2100F, JEOL, Ltd.). The elemental compositions of samples were analyzed using energy-dispersive analysis of X-ray (EDAX), which was connected with TEM. ImageJ software was used to measure the diameter distribution of each sample. The Fourier transform infrared spectra of the nanofibrous scaffolds were tested on a Nicolet 8700 FTIR spectrometer (Thermo Nicolet Ltd., United States) to study the functional groups in a range of 400–4,000 cm^−1^ at room temperature. Thermal gravimetric analysis was used by TG209F1 (NETZSCH-Gerätebau GmbH, Germany) to evaluate the thermal parameters from the produced scaffolds.

### Porosity

The porosity of each scaffold was analyzed *via* the liquid intrusion technique (O'Connor et al., 2021). The specific samples were measured and immersed in 100% ethanol for 24 h under agitation to ensure complete wetting. Then the samples were taken out, and excess fluids were removed from the surface. The porosity was calculated by the following formulas:

$${\text{V}}_{\mathrm{scaffolds}}=\;\frac{m_{\mathrm{dry}}}{\rho_{\mathrm{scaffold}}}$$

$$V_{\mathrm{EtOH}}=\frac{m_{\mathrm{wet}}-m_{\mathrm{dry}}}{\rho_{\mathrm{EtOH}}}$$

$$\text{Porosity}=\frac{V_{\mathrm{EtOH}}}{{\text{V}}_{\mathrm{scaffolds}}-V_{\mathrm{EtOH}}}\times 100\%,$$

where ${\text{V}}_{\mathrm{scaffolds}}$ is the volume of each scaffold and $V_{\mathrm{EtOH}}$ is the volume of ethanol absorbed by the scaffolds. $\;m_{\mathrm{dry}}$ and $m_{wet}$ refer to the dry and wet weights of scaffolds. $\rho_{EtOH}$ = 0.789 g cm^−3^ and $\rho_{\mathrm{scaffold}}$ = 1.20 g cm^−3^, calculated by the ratio 4:1 between the density of PLA (1.25 g cm^−3^) and PCL(1.02 g cm^−3^).

### Mechanical Properties

Mechanical properties were measured by TMA Q400 (TA Instruments, United States) on rectangular-shaped specimens with 7 mm length and 6 mm width. The representative stress–strain curves were reported for each sample.

### Biodegradation Analysis

The biodegradation analysis of nanofibrous scaffolds was performed in 10 ml of phosphate-buffered saline (PBS, pH = 7.4) at 37°C for 7 days. The weight of each sample was recorded before and after incubating in PBS solution at a specific time. The degradation of the scaffolds was calculated as follows:

$$\text{Weight}\;\text{loss}\left(\text{\%}\right)=\frac{W_{0}-W_{t}}{W_{0}}\times 100\%,$$

where W_0_ and W_t_ refer to the mass before and after incubation, respectively.

### Drug Loading Efficiency and Release Studies of Multilayered Nanofibrous Scaffolds

For drug loading efficiency, the drug-loaded PVA nanofiber samples were immersed in 50 ml deionized water precisely to get clear solutions. The drug loading efficiency was calculated by the following equation:

$$M_{L}=\frac{M_{A}}{M_{T}}\times 100\%,$$

M_L_ is the loading efficiency, M_A_ is the actual concentration of drug measured by UV–visible spectrophotometer (UV2600, SHIMADZU, Japan), and M_T_ is the theoretical concentration of drug calculated from drug/nanofiber ration. The characteristic absorption peak at 294 and 254 nm was monitored for MH and SSD, respectively.

To analyze the release behavior of drug from the multilayered scaffolds, the initial weight of the samples was measured. Then, all samples were incubated in 10 ml PBS solution at 37°C. After the specific time, 1 ml of solution was taken out from the vials, and an equal volume of fresh PBS solution was replenished. The release curves were evaluated by using a UV-vis spectrophotometer.

### Contact Angle

The water contact angle of nanofibrous scaffolds was evaluated by a video contact angle instrument. A drop of distilled water was cast on the surface of nanofibrous scaffolds. A tangent line of the intersection of the droplet and the surface was drawn to measure the contact angle values. For each test, three estimations were performed in a different area.

### Antibacterial Activity Tests

The antibacterial activity of antibiotic-loaded nanofibrous scaffolds against *Staphylococcus aureus* and *Escherichia coli* was determined by a disc diffusion assay. In brief, *S. aureus* and *E. coli* suspension with a concentration of 10^6^ CFU/ml was spread with a swab onto nutrient agar plates, respectively. The scaffolds were cut into 6-mm-diameter discs with a mass of 1–3 mg and deposited in each plate. Samples were incubated at 37°C, and the inhibition zones were observed and measured precisely after 18 h.

## Results and Discussion

### Surface Morphology and EDAX Analysis of Composite Nanofibrous Scaffolds

**FIGURE 1:**
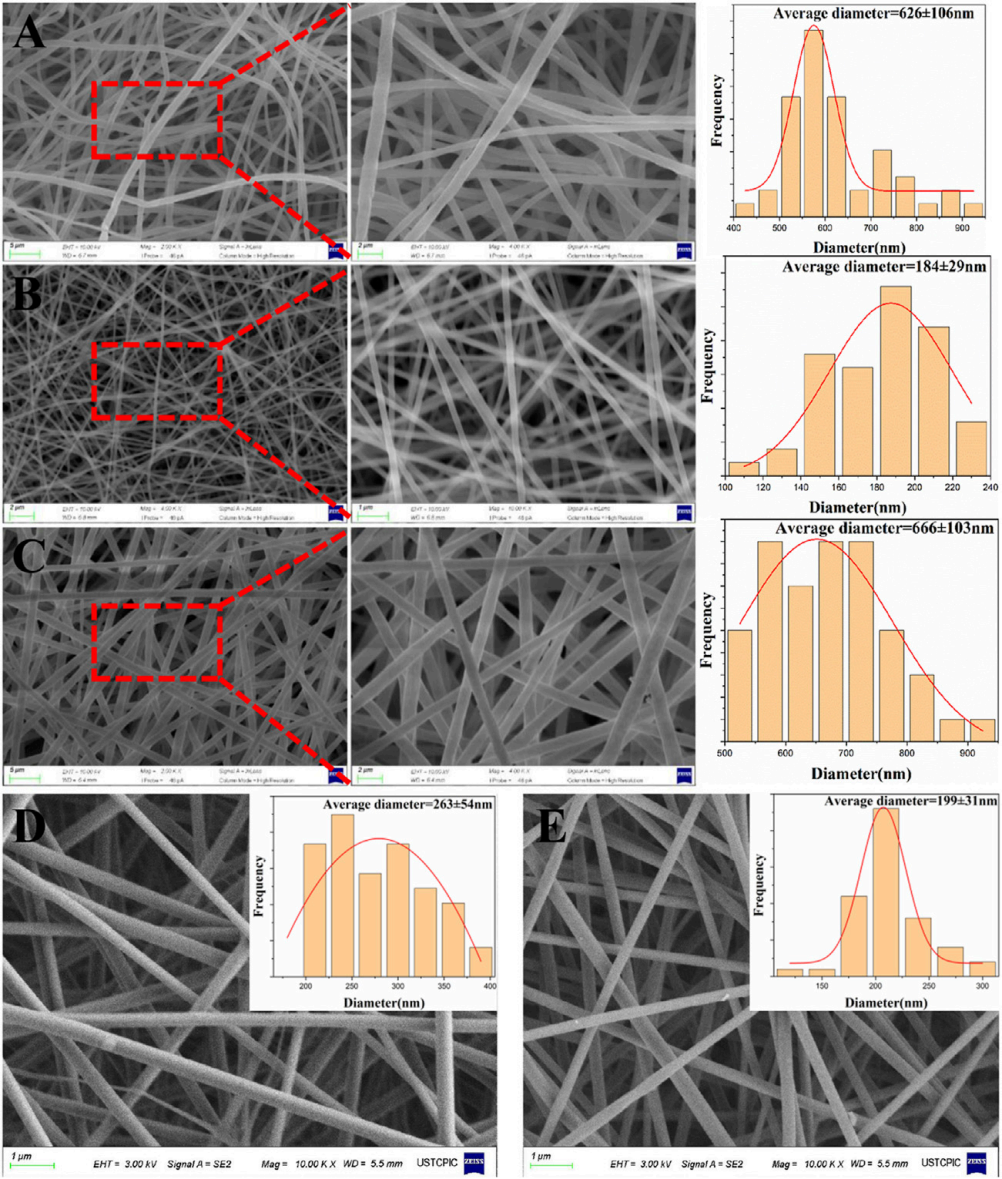
SEM images and diameter distribution of PLA: PCL **(A)**, PVA **(B)**, ML-S **(C)**, PVA:MH **(D)**, and PVA:SSD **(E)**.

By changing the applied voltage, the concentration of the solution, and other parameters during the electrospinning process, nanofibers with different morphologies can be manufactured. In the preliminary experiment, we explored and selected the proper parameters. Figure 1 shows the SEM images of PLA: PCL nanofibers, PVA nanofibers and multilayered nanofibrous scaffolds (ML-S), and drug loading nanofibers. It can be seen that the surface of multilayered scaffolds is covered with PLA:PCL nanofibers. The consistency between A and C shows that the properties of nanofibers have not changed in multilayered electrospinning. The average diameter of PVA nanofibers is smaller than that of PLA:PCL nanofibers. The reason is the greater viscosity and lower conductivity of the PLA:PCL solution, compared with the PVA solution (Xue et al., 2019). The high viscosity of the solution limits the stretching of the jet during spinning, which is conducive to the formation of larger diameter. On the contrary, the high conductivity of the solution has also been proven to promote diameter reduction (Deshawar et al., 2020). The design of different diameters is to ensure that the outer layer has a good encapsulation effect and a high surface area ratio of drug loading layer, in order to facilitate the delivery and release of the drug.

For drug loading layer, MH and SSD were selected as model drugs. The scaffolds were manufactured by blending and suspension electrospinning and evaluated for performance respectively. It can be seen from Figures 1D, E that the morphology of drug loading nanofibers has not changed significantly. The diameter of PVA/MH nanofiber increased to 263 ± 54 nm compared with blank PVA nanofiber, which is consistent with previous studies (Giram et al., 2018). There are some clumps—probably insoluble drugs—on the surface of the nanofiber produced by suspension electrospinning (Coimbra et al., 2019). The diameter was roughly the same as the PVA nanofiber, with only a little increase.

**FIGURE 2:**
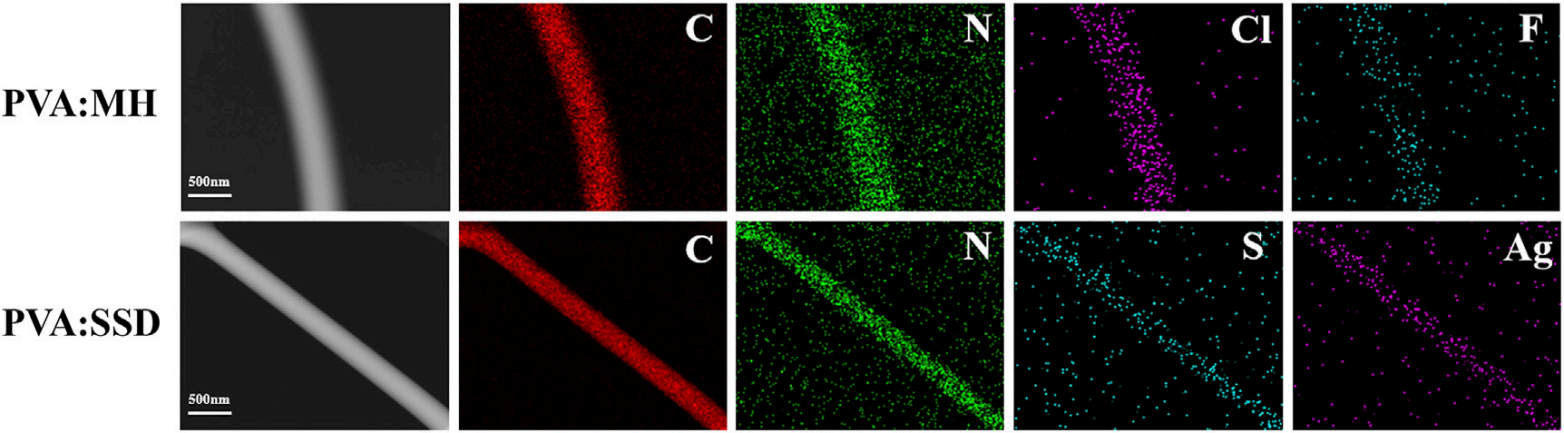
TEM images and the elemental mapping images of PVA: MH and PVA:SSD.

**FIGURE 3:**
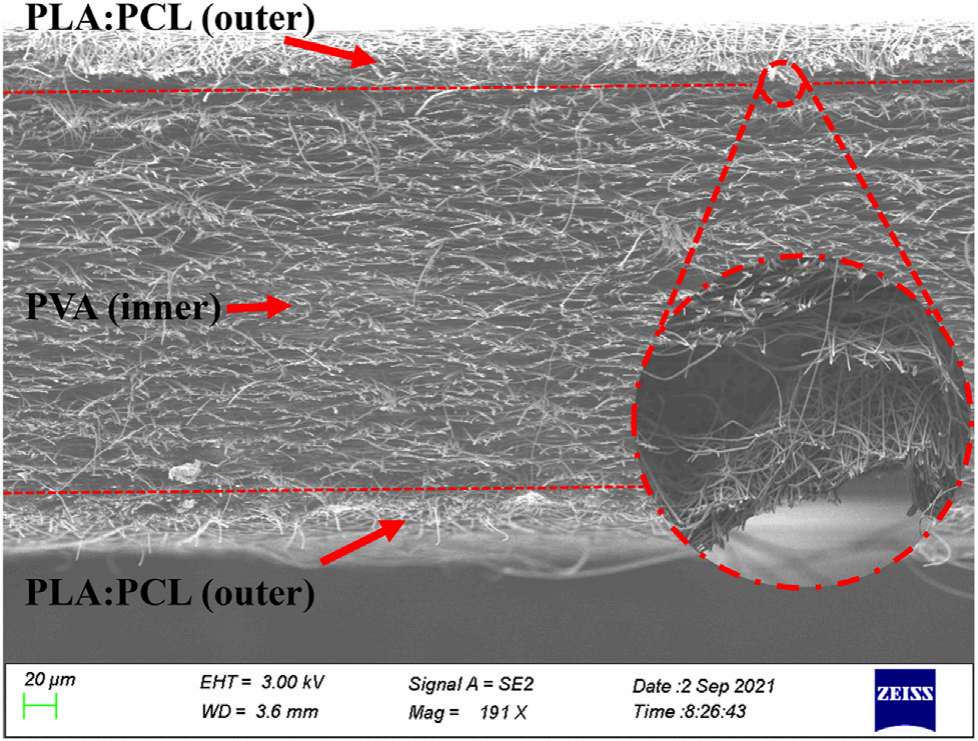
Cross-section image of ML-S; the enlarged part is the interface details.

The presence of MH and SSD loaded in PVA nanofibers was further confirmed by the TEM and EDAX elemental mapping images, as shown in Figure 2. The mapping images indicate the existence of specific element contained in MH (N, Cl, F) and SSD (N, S, Ag), which is not a constituent element of PVA. A cross-section image (Figure 3) of ML-S demonstrated that the multilayered structure is composed of two outer layers of PLA:PCL nanofibers and an inner layer of PVA nanofibers. The different layered regions have been marked in Figure 3 using arrows. The thickness of PVA is about 7 times (230:30 µm) that of one-sided PLA:PCL. The dimensions of different layers can be adjusted by modifying the electrospinning parameters, which also affect the release performance and degradation of the multilayered scaffolds.

### Characterization of Each Sample

**FIGURE 4:**
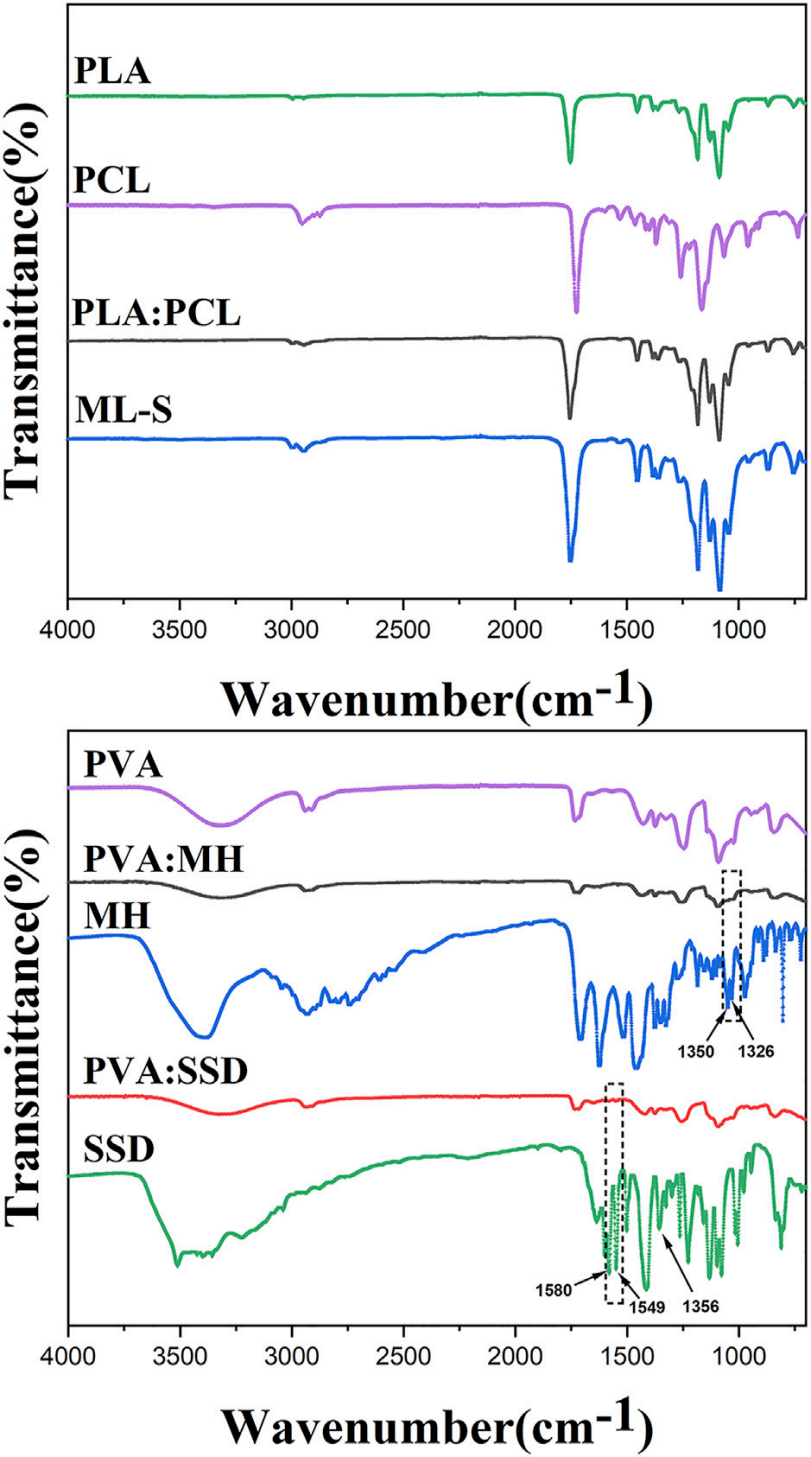
FTIR spectra of PLA, PCL, PLA: PCL, ML-S, PVA, MH, SSD, PVA: MH, and PVA:SSD.

In order to confirm that the surface of ML-S prepared by blending electrospinning method forming a homogeneous chemical structure, ATR-FTIR spectra were carried out for each sample and showed their typical functional group, as listed in Figure 4. For a pure PLA nanofiber, the typical peaks at 2,945–2999,1454, 1,265, and 1,045 cm^−1^ were attributed to –CH- stretch, -CH_3_ bend, -C-O- stretch, and -OH bend (Mohandesnezhad et al., 2020). The peaks at 2,952, 2,871, and 1,065 cm^−1^ showed an asymmetrical stretch of –CH2 and symmetrical elongation of –CH2 and -C-O of pure PCL nanofiber. Furthermore, carbonyl stretch of –C=O was observed at 1752 cm^−1^ and 1724 cm^−1^ of PLA and PCL, respectively (Scaffaro et al., 2017). The same characteristic peaks also appear in PLA:PCL and ML-S samples, indicating that the uniform hybrid was formed during the blending electrospinning process. The broad peak at 3,318 cm^−1^ was associated with -OH stretching of a PVA nanofiber, owing to the strong interaction of the hydrogen bond. The abundant hydrogen bonds of PVA lead to excellent drug loading performance and encapsulation efficiency (Ahmadi Majd et al., 2016). The peaks at 2,910, 1734, and 1,092 cm^−1^ showed –CH_2_ stretch, -C=O, and -C-O-C- groups of PVA nanofiber (Hulupi and Haryadi 2019). The detailed characteristic peak analysis is listed in Table 2 (Pattanashetti, Achari, Torvi, Doddamani, and Kariduraganavar 2020).

**TABLE 2:** Main infrared bands in the FTIR (ATR) in the region from 4,000 cm^−1^ to 700 cm^−1^.

Polymer	Wavenumber (cm^−1^)	Assignment
PLA	2,999 (asymmetric); 2,945 (symmetric)	-CH- stretch
—	1,752	-C=O carbonyl stretch
—	1,454	-CH_3_ bend
—	1,382 (asymmetric); 1,362 (symmetric)	-CH- deformation
—	1,265	-C-O- stretch
—	1,208	-C=O bend
—	1,181; 1,129; 1,086	-C-O- stretch
—	1,045	-OH bend
—	955	-CH_3_ rocking mode
—	868	-C-C- stretch crystalline phase
PCL	2,952	-CH_2_ stretch, asymmetrical
—	2,871	-CH_2_, elongation, symmetrical
—	1,724	-C=O carbonyl stretch
—	1,065	-C-O
PVA	3,318	-OH stretch
—	2,910	-CH_2_ stretch
—	1,734	-C=O
—	1,374	-CH_2_
—	1,326	-C-H and –OH bending
—	1,092	-C-O-C-

For MH-loaded nanofibers, the C-F group was represented by the peaks at 1,350 cm^−1^ and 1,326 cm^−1^ (Fu et al., 2016), which were witnessed in the spectrum of MH and PVA:MH. The characteristic peaks of SSD also appear in PVA:SSD nanofiber, for example, vibrational stretching of its phenyl structure conjugated to the NH_2_ group at 1,580 cm^−1^ and the phenyl group at 1,549 cm^−1^, confirming the presence of SSD loaded in the PVA nanofibers (Li et al., 2016; Semnani et al., 2018). The reason why some characteristic peaks are not obvious is due to the low content of SSD (Alipour et al., 2019), such as the band at 1,356 cm^−1^ (asymmetrical stretching of the S=O bonding). In brief, the analysis of FTIR showed that antibiotics were successfully loaded on the nanofiber through electrospinning.

**FIGURE 5:**
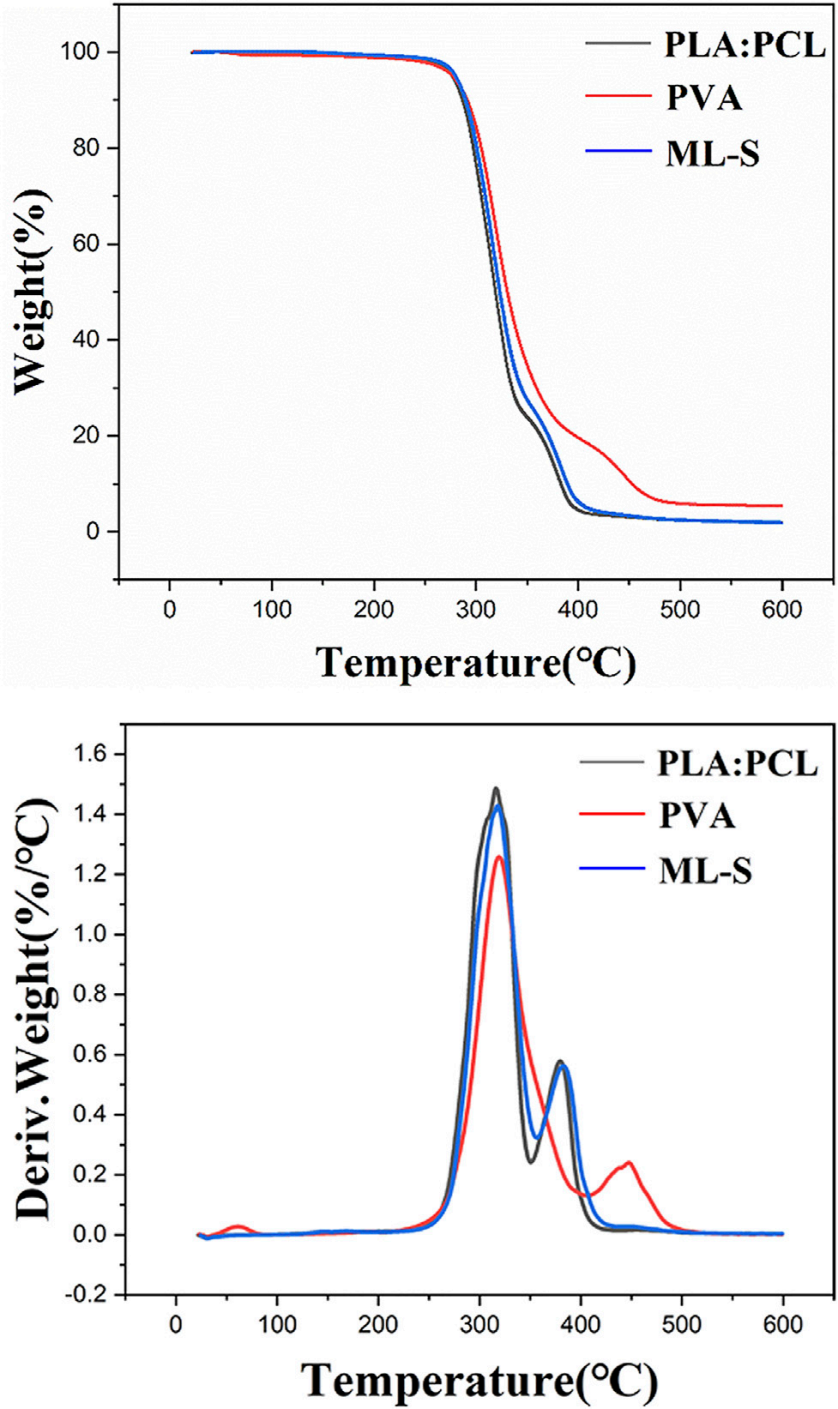
TG and DTG profiles of PLA:PCL, PVA, and ML-S.

Thermal gravimetric analysis of the sample was tested by heating from room temperature to 600°C degrees under nitrogen atmosphere, and the TG curves were displayed in Figure 5. For PLA:PCL, the weight loss stage takes place over 200°C, and the whole mass loss is about 97%. All samples present two successive weight loss stages during the heating process. The presence of two distinct degradation temperatures in TG curves of PLA:PCL is reflected in the presence of thermodynamic immiscibility between two phases (PLA and PCL) in the blends (Herrero-Herrero et al., 2018; Sharma and Satapathy 2019). The first stage of PVA is in the temperature of 202–402°C, with loss of 79.4%, which is attributed to the breakage of the side chain of PVA (Hang et al., 2010). The weight loss of the second stage less than 505°C is about 21.8%, which is due to the thermal breaking of weak bonds in the polymeric structure (Wu, Ooi, Song, Wang, Liu, Lin, Chiu and Chang 2021). Two distinct peaks in DTG curves represent the temperatures where there is a noticeable change in weight loss rate during the decomposition. By comparison to TG curves, it is believed that the properties of each layer were not affected in ML-S through multilayered electrospinning.

### Hydrophilicity and Porosity of Scaffolds

**FIGURE 6:**
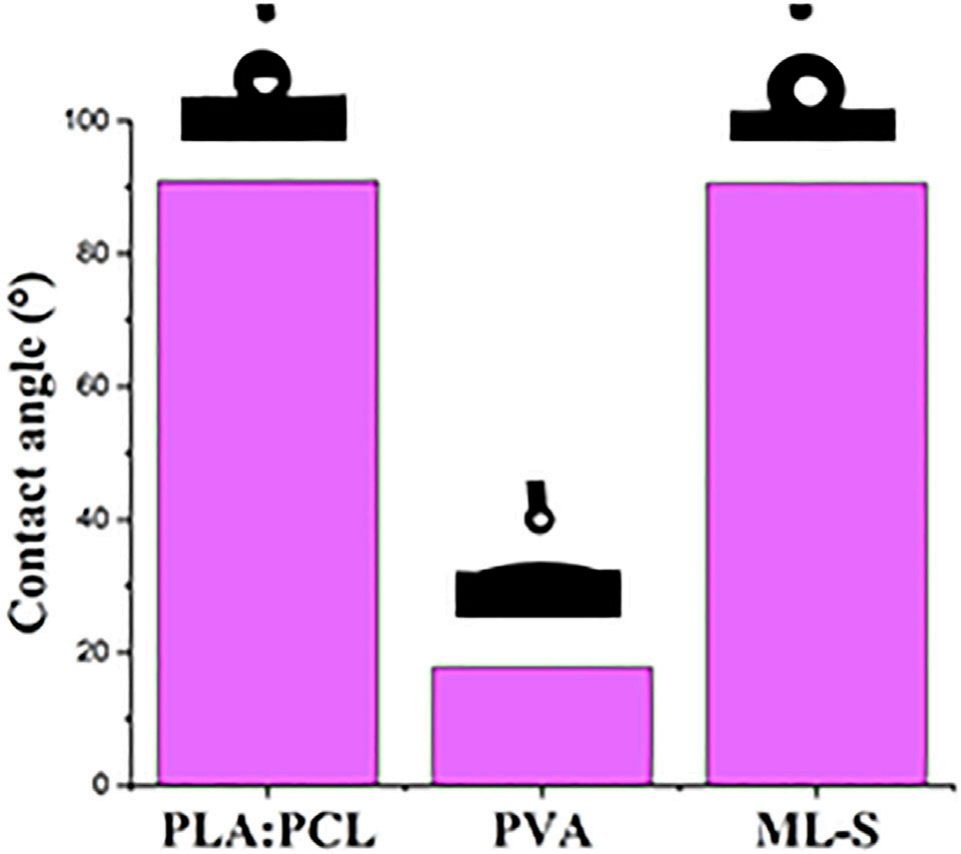
Water contact angle of PLA:PCL, PVA, and ML-S.

The hydrophilicity of all samples was evaluated by the contact angle of the water drop deposited on the surface of the nanofibers. The results are listed in Figure 6. PVA nanofibers are more hydrophilic than the other samples, owing to abundant hydroxyl groups in the structure (Abdal-Hay et al., 2016). PLA:PCL nanofiber and ML-S showed obvious hydrophobic properties with *θ* ≈ 91°, which is the same as previous studies (Sharma and Satapathy 2019). The hydrophilicity of PVA means quick degradation after contact with water. Thus, the resulting abrupt release of drug loaded is not suitable for long-term treatment in certain medical situations. The presence of hydrophobic surfaces allows the multilayered scaffold to continuously release drugs for a long time and induce self-cleaning properties for potential application (Jamaluddin et al., 2021).

One of the advantages of electrospinning is the high specific surface area ratio and porosity. For the drug delivery system, the specific surface area affects the encapsulation rate and release rate of the drug (Kalantari et al., 2019). Past studies have shown that the porosity of the scaffold is higher than 77%, which is suitable for the infiltration and proliferation of fibroblasts (Chong et al., 2007; Pedram Rad et al., 2019). The estimated porosities of all the samples are presented in Table 3. All formulations showed high porous, which is the same as the typical values reported in literature (Rafiei, Jooybar, Abdekhodaie and Alvi 2020; O'Connor, Cahill and McGuinness 2021). PVA nanofiber has higher porosity (92.8%) due to smaller diameter, compared to PLA:PCL sample (76.9%). As a superposition of PLA:PCL and PVA, the porosity of ML-S is between the two samples. In the case of acute injury, the wound needs to be treated quickly to prevent bacterial infections associated with the wound. Nanofibers with high specific surface area and porosity can absorb biological fluids and prevent microorganisms from penetrating deep wounds.

**TABLE 3:** Characterization of each sample.

Sample	Porosity (%)	Contact angle (◦)	Young’s modulus (MPa)	Tensile strength (MPa)	Strain at break (%)
PVA	92.8	17.78	74.5 ± 0.22	7.62	103.2
PLA:PCL	76.9	90.96	22.3 ± 0.10	2.03	241.3
ML-S	89.6	90.61	27.1 ± 0.19	2.53	176.4

### Mechanical Properties

**FIGURE 7:**
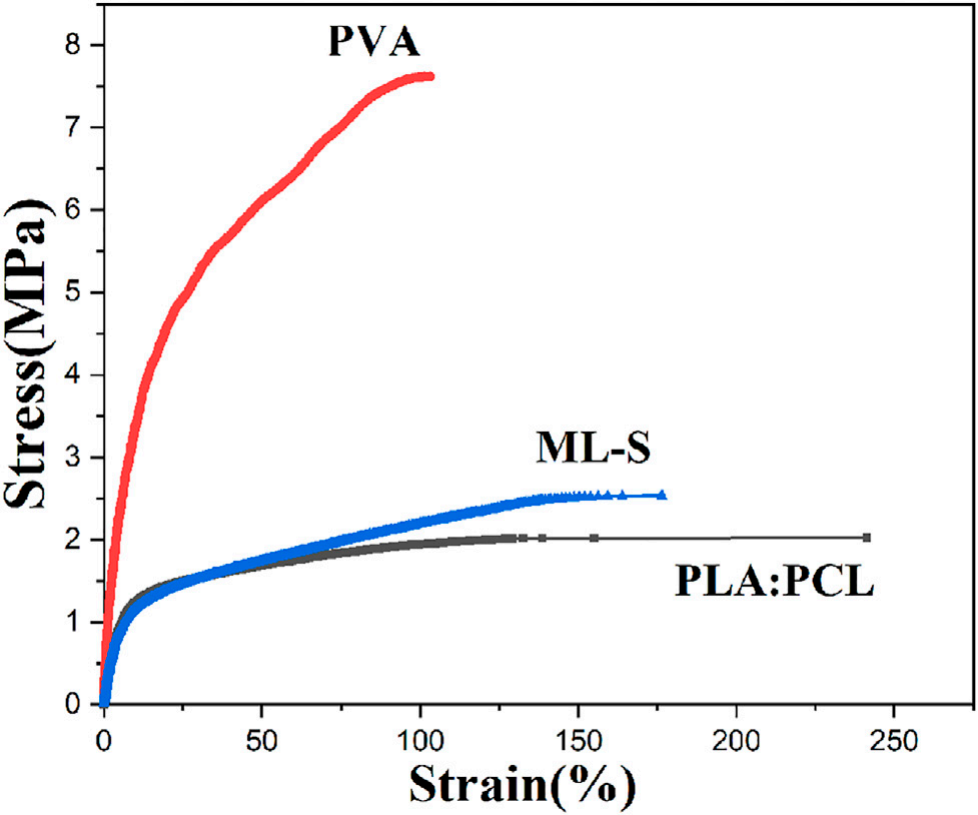
Stress–strain curves of PLA:PCL, PVA, and ML-S.

The tensile properties of various samples under uniaxial tension were treated using the stress versus strain profile shown in Figure 7 and Table 3. The mechanical properties of the samples are affected by the different formulas of the polymer solution. Biomedical materials must have a certain degree of mechanical strength to deal with deformation, damage, tearing, etc. in different environments (Vilchez et al., 2020). Since the tensile strength of skin was reported as 1–40 MPa, all samples were suitable for wound dressing (Sander et al., 2014). Combined with the results of SEM, the decrease in average nanofiber diameter led to an increase in tensile strength owing to the aligned structure instead of bulk properties (Eren Boncu and Ozdemir 2021). The reason why diameters affect mechanical properties was that fibrous structure and high molecular orientation become denser with the diameter being decreased (Castkova et al., 2020).

With the addition of PVA, Young’s modulus and tensile strength of ML-S increased, but the toughness was relatively poor. The uniform dispersion of PVA nanofiber to ML-S through multilayer electrospinning leads to the increase of mechanical performance, which is also explained by the differences between PLA:PCL and PVA. Briefly, the mechanical properties of ML-S are moderate among all the samples.

### *In Vitro* Biodegradation Studies

**FIGURE 8:**
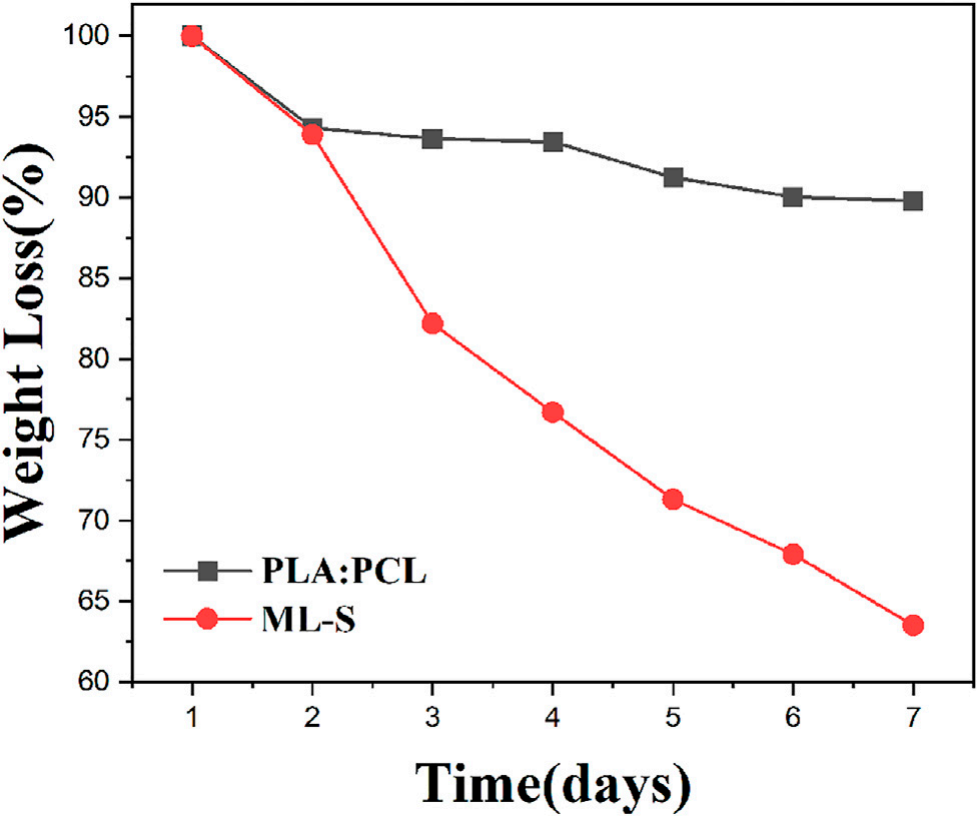
Biodegradation study of PLA:PCL and ML-S.

The biodegradation studies of the different samples were carried out by incubating samples in PBS of pH = 7.2 for a week. The rate of degradation was calculated in terms of mass loss percent and the results are illustrated in Figure 8. From day 1 to day 7, it was observed that the rate of degradation roughly decreased. The mass of PLA:PCL is relatively stable; only a less than 10% reduction has been shown, whereas ML-S exhibited a higher degradation rate due to the existence of a hydrophilic PVA nanofiber layer. The curve dropped sharply in the first 3 days and then became flat. Seven days later, the mass loss of ML-S reached about 37%.

The results showed that the degradation of all samples is fast in the early stage and slow in the later stage. Related research has demonstrated the degradation rate of PCL is slower, compared to PLA (Pattanashetti, Achari, Torvi, Doddamani, and Kariduraganavar 2020). Considering good biocompatibility and biodegradability, we selected the mixture of PLA and PCL as the outer layer of the drug delivery system, instead of pure PCL. The lower rate of degradation for PLA:PCL nanofibers is attributed to the compact structure and hydrophobicity. During the preparation process of ML-S, the mass ratio of PLA: PCL to PVA is controlled at about 2:1. According to the mass ratio of each component, we inferred that the most mass loss of ML-S came from the degradation of PVA, which is beneficial to drug release.

### Drug Loading Efficiency and Release Studies

The drug loading efficiency of MH and SSD in the optimized PVA nanofibers was 93.3 and 70.7%, respectively. The higher MH loading efficiency might be ascribed to its hydrophilicity, compared to SSD (Nalbandi and Amiri 2018; Hameed et al., 2020). The clearness suggested that MH and PVA were homogeneously dissolved in the precursor solution. However, poor water solubility caused SSD to be present as a nonuniform dispersion of nanoparticles on the nanofiber surface, which was observed by SEM (Alipour, Khorshidi, Shojaei, Mashayekhi and Moghaddam 2019; Coimbra, Freitas, Goncalves, Gil and Figueiredo 2019).

**FIGURE 9:**
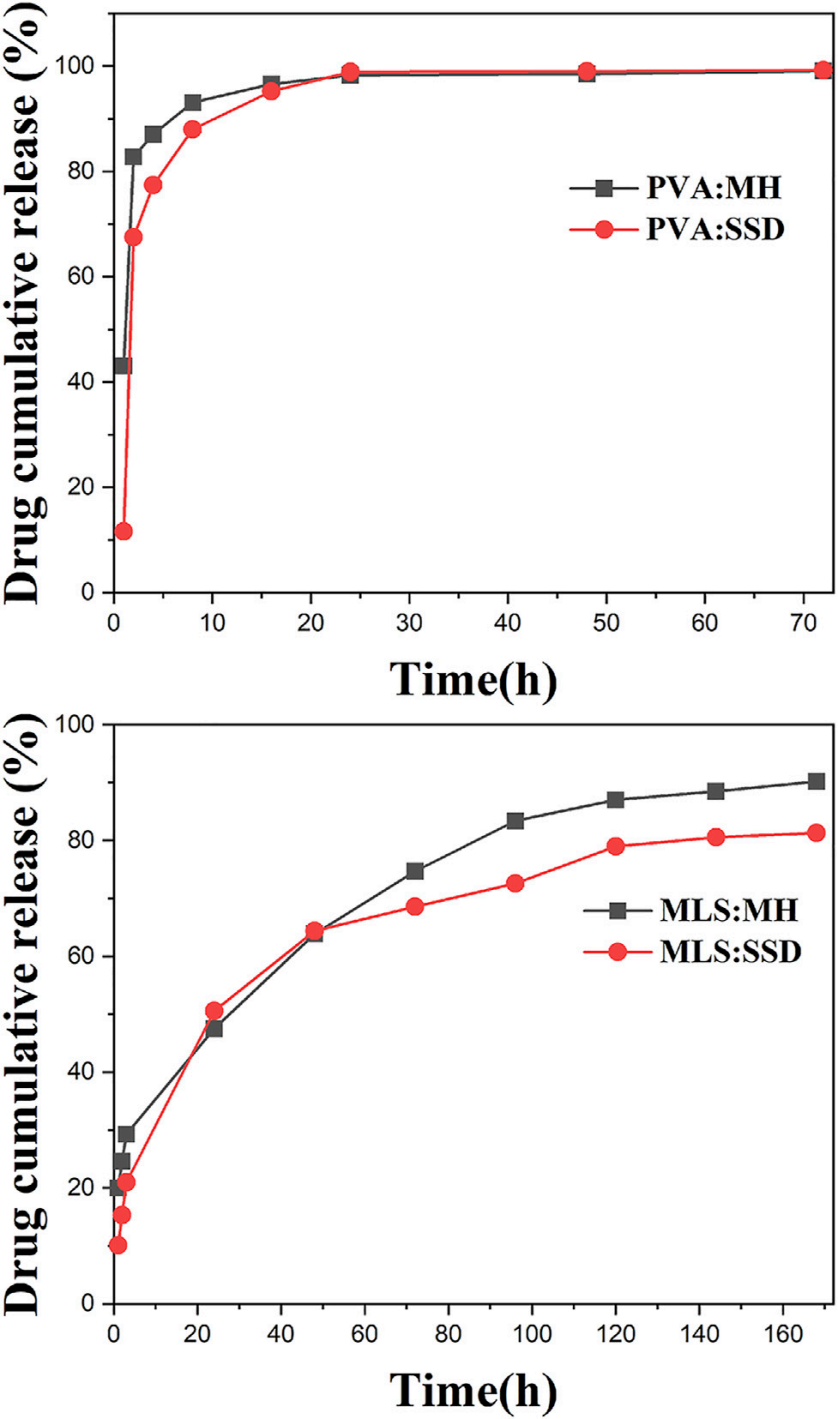
Drug release curves of PVA:MH, PVA:SSD, ML-S:MH, and ML-S:SSD.

The drug release performance of a drug delivery system affects its efficacy for various biomedical applications. The suffering of patients could be reduced by the control of the drug release over a period since frequent dressing changes can be eliminated. The drug release curves are shown in Figure 9. For PVA nanofibers, a burst release of 80–90% of the drug was observed for both PVA:MH and PVA:SSD, and the remaining drug is completely released within 24 h. The reason is the rapid degradation of PVA nanofibers without cross-linking treatment during the entire preparation. On the other hand, the multilayered nanofibers scaffolds exhibited excellent continuous drug release performance. After 1 day, approximately 47.5 of MH and 50.6% of SSD were released from ML-S:MH and ML-S:SSD. The entire drug release process is maintained within 7 days. The rate of drug release from the ML-S was slower, which could be attributed to the fact that the drug was loaded in the inner layer and protected by the outer. There is no significant difference in the release performance between the two drugs for the same drug delivery system. The slightly lower content of SSD may be due to hydrophobicity, which also affects its antibacterial properties. In addition to the diffusion of the antibiotic component from the nanofibers into the surrounding solutions, polymer degradation (mainly PVA) also contributes to the drug release, which is consistent with the results of degradation studies (Gong et al., 2018). By the results of release curves, we inferred that the time interval expected during which scaffolds provide antibacterial activity is about a week. Past studies have shown that most of the release curves have a burst release in the initial stage and then become moderate gradually. The release interval of multilayer scaffolds is mostly distributed in 4 h to 1 week, which is affected by the preparation process and materials (Cerqueira et al., 2016; Laha et al., 2017; Pedram Rad et al., 2019; Charpashlo, Ghorani and Mohebbi 2021). The initial burst release is sometimes necessary especially for the fast effectiveness of the formulations in the case of infection. The subsequent recovery stage needs sufficient drug concentration for antibacterial effect. Therefore, it is reasonable to believe that ML-S has a gradual and controlled release behavior and provides a desirable repair effect for wound treatment.

### *In vitro* Antibacterial Activity

**FIGURE 10:**
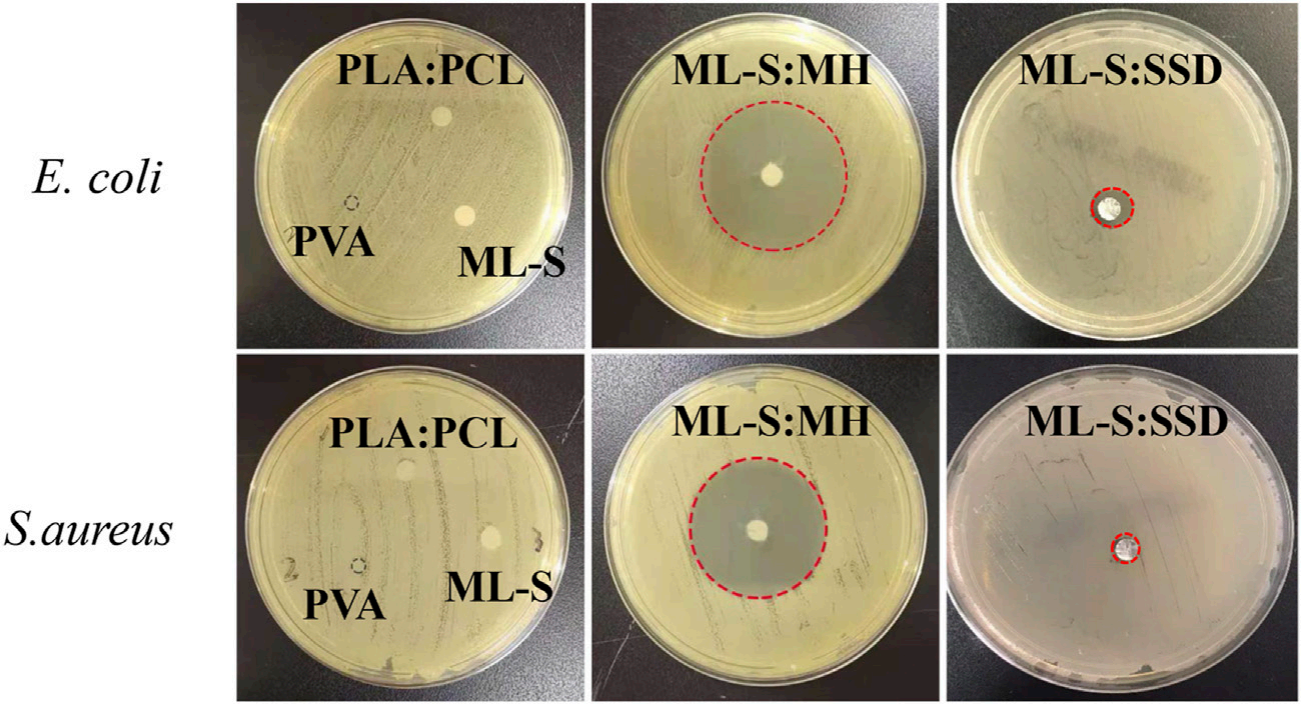
Antimicrobial activity of PLA:PCL, PVA, ML-S, ML-S:MH, and ML-S: SSD.

The antibacterial assay was assessed by incubating prepared samples in an agar disc. MH and SSD were used as model drugs and loaded with ML-S through the blending and suspension electrospinning method. Those nanofibers or scaffolds were exposed to the area filled with a specific amount of microbial (Gram-positive and Gram-negative, respectively). After 18-h incubation, the zone of inhibition was shown on the plates (Figure 10). Neither PLA:PCL nor PVA nanofibers exhibited antibacterial activity. An obvious zone of inhibition was observed at the plate of both ML-S:MH and ML-S:SSD, which confirmed that antimicrobial performance is not affect by different electrospinning methods. Coimbra et al. reported assembling antibiotics with nanofibers by suspension spinning (Coimbra, Freitas, Goncalves, Gil and Figueiredo 2019). However, the increase in the MH inhibition zone is due to the more significant antibacterial properties and hydrophilicity, with SSD (Keating and Scott 2004; Li, Wu, Li, Zhao and Qu 2016). As shown in the earlier SEM results, part of the undissolved SSD formed clumps on the nanofiber surface. The uneven dispersion may affect the release of the drug, which could be a target for future research.

Bacterial infections are associated with many medical situations, resulting in slower wound recovery, fever, and other complications (Ibrahim et al., 2021; Karmakar 2021). Therefore, antibacterial activity is necessary for biomaterials. The results indicated that the scaffold-loaded drug displayed good antibacterial activity aim at both Gram-positive and Gram-negative bacteria.

## Conclusion

Multilayered nanofibrous scaffolds containing PLA and PCL were fabricated using electrospinning, and the morphological, thermal, mechanical, hydrophilic, biodegradable, and release properties were analyzed. SEM results showed that beadless and uniform nonwoven nanofiber were produced on the surface with a range from 500 to 900 nm. TEM and element analysis exhibited the existence of antibiotics in nanofibers. The scaffolds are thermally and mechanically stable for medical application. Hydrophobic surface and hydrophilic inner layer ensure satisfactory drug delivery performance, as well as good biodegradation, and the mass loss for degradation reached 37% in 7 days. The current drug release interval is about 1 week, which may be extended by cross-linking treatment for the inner PVA nanofibers. The antibacterial application proves that the compatibility of the drug and the carrier needs to be considered in order to achieve good performance of the drug delivery system. The nanofibrous scaffolds with loaded MH exhibited excellent antimicrobial performance against both of the gram-positive *S. aureus* and gram-negative *E. coli* bacteria. Therefore, multilayered nanofibrous scaffolds present an appropriate method for drug delivery system with preservation of biological activity of the loaded substance. These drug-loaded scaffolds may serve as a promising engineered system for wounding dressings, tissue engineering, or other biomedical applications.

## Data Availability

The original contributions presented in the study are included in the article/supplementary material; further inquiries can be directed to the corresponding author.
